# Hypocholesterolemic Effect of Blackcurrant (*Ribes nigrum*) Extract in Healthy Female Subjects: A Pilot Study

**DOI:** 10.3390/molecules26134085

**Published:** 2021-07-04

**Authors:** Naoki Nanashima, Kayo Horie, Maiko Kitajima, Shizuka Takamagi, Kasumi Mikami, Naoya In, Toshiko Tomisawa

**Affiliations:** 1 Department of Bioscience and Laboratory Medicine, Hirosaki University Graduate School of Health Sciences, Hirosaki 036-8564, Japan; k-horie@hirosaki-u.ac.jp; 2 Department of Nursing Sciences, Hirosaki University Graduate School of Health Sciences, 66-1 Hon-cho, Hirosaki 036-8564, Japan; kitajima@hirosaki-u.ac.jp (M.K.); takamagi@hirosaki-u.ac.jp (S.T.); k-mikami@hirosaki-u.ac.jp (K.M.); in1105@hirosaki-u.ac.jp (N.I.)

**Keywords:** atherosclerosis, cholesterol, lipoprotein, serum lipid

## Abstract

Blackcurrant extract (BCE) ameliorates dyslipidemia in menopausal model animals and in elderly women at a risk of dyslipidemia. However, it is unknown whether the daily intake of BCE can prevent lipid abnormalities in healthy individuals. Lipids are essential for the body, but they also cause arteriosclerosis. In this noncomparative pilot study, we examined the effects of BCE administered for 29 days on serum lipids in young healthy women. Blood samples were collected before and on days 4 and 29 after BCE intake, and 20 lipoprotein fractions in the serum were separated using a gel-permeation high-performance liquid chromatography method to measure the triacylglycerol and cholesterol levels in lipoproteins. There were no effects on lipids on day 4 of BCE intake, but the total cholesterol level decreased on day 29. Furthermore, the levels of total very-low-density lipoprotein (VLDL) cholesterol, small VLDL cholesterol, and large low-density lipoprotein cholesterol were significantly decreased. These results suggest that the daily intake of BCE has a hypocholesterolemic effect in healthy women, and that it is effective in preventing atherosclerosis.

## 1. Introduction

Blackcurrant (*Ribes nigrum* L.) is a shrub grown in cooler regions of Europe and New Zealand for its dark-purple berries, which are approximately 1 cm in diameter. The berries are rich in anthocyanins, a type of polyphenol present in pericarp, and essential fatty acids, such as γ-linolenic acid, in seeds, which are known to have various health-promoting functions, including strong antioxidant and phytoestrogenic effects, and blood flow-improving ability [1,2,3,4]. Lipids are water-insoluble organic substances found in cells and blood. Several types of lipids are synthesized in the body, but many others are obtained from food. Most of the lipids in human blood bind to proteins, forming water-soluble lipoproteins that flow in the blood. Lipids are metabolized as cell membrane constituents, energy sources, and materials used in the synthesis of hormones and other bioactive compounds. Thus, lipids are essential for the body. However, they have garnered attention as causative agents of arteriosclerosis and cardiovascular diseases [5,6,7,8].

The structure of lipoproteins comprises a nucleus made of hydrophobic lipids such as triacylglycerols (TG) and cholesterol esters, which is surrounded by an outer shell of amphipathic lipids such as phospholipids and free cholesterol. Lipoproteins are classified into the following five types according to their specific gravity: chylomicron (CM), very-low-density lipoprotein (VLDL), intermediate-density lipoprotein (IDL), low-density lipoprotein (LDL), and high-density lipoprotein (HDL) [9,10].

CM is the largest lipoprotein in the blood and carries food-derived TGs. VLDL is a TG-rich lipoprotein that is synthesized in the liver. It becomes an IDL under the action of lipoprotein lipase. The hepatic triglyceride lipase converts most of the IDLs to LDLs, which are taken up by the LDL receptor and catabolized. LDL is the major carrier of cholesterol. In addition, it is the final metabolite of VLDL and is taken up by the LDL receptor present in hepatocytes and peripheral tissues. HDL extracts cholesterol from various tissues and transports it to the liver [11,12].

Polyphenols present in some plants are known to ameliorate dyslipidemia [13,14]. It has been reported that the administration of blackcurrant extract (BCE) to animals fed a high-fat diet improved dyslipidemia, and that BCE even lowers serum lipids in humans at risk of dyslipidemia [15,16,17,18]. Previously, we showed that BCE is effective in improving menopausal dyslipidemia [19]. This suggests that the daily intake of BCE in healthy subjects may prevent dyslipidemia. However, the effects of BCE on serum lipids in healthy humans have not been investigated. Moreover, a detailed study to examine its effect on 20 lipoprotein fractions separated from serum using a gel-permeation (GP) high-performance liquid chromatography (HPLC) method has not been undertaken. Therefore, we conducted this noncomparative pilot study to investigate the effects of BCE, administered to healthy women without dyslipidemia for approximately 1 month, on serum lipids.

## 2. Results

### 2.1. Serum Triglyceride and Total Cholesterol Content

**Figure 1:**
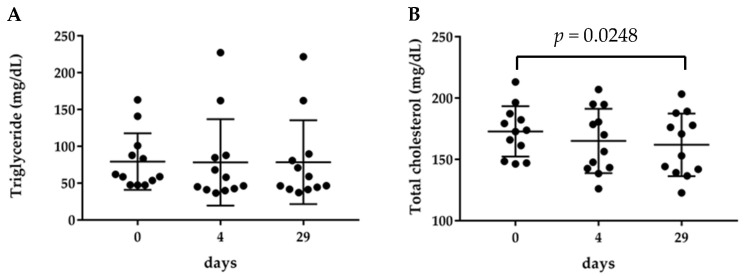
Changes in the levels of serum lipids. Level of (**A**) triglyceride and (**B**) total cholesterol. The data are presented as individual data with median and range, *n* = 12.

Blood was collected before and 4 and 29 days after the intake of BCE, to separate serum. The levels of TG and total cholesterol in the serum were measured. Triglyceride levels did not change before and after the intake of BCE (Figure 1A). On the contrary, the total cholesterol level was 172.8 ± 20.6 mg/dL before BCE intake. This did not change 4 days after BCE intake, but significantly decreased to 161.9 ± 25.5 mg/dL 29 days after the intake (Figure 1B).

### 2.2. Lipoprotein Profiling

The total cholesterol in the serum decreased 29 days after the intake of BCE; therefore, we investigated the levels of cholesterols in lipoproteins. There were no differences between the levels of CM and HDL cholesterols. The LDL cholesterol level tended to decrease on days 4 and 29 after BCE intake, but no significant difference was observed. However, the VLDL cholesterol level significantly decreased from 24.4 ± 7.2 mg to 18.6 ± 4.7 mg after the intake of BCE (Table 1). In contrast, the TG level did not change with any lipoprotein (Supplementary Materials, Table S1).

### 2.3. Lipoprotein Detail Profiling

Lipoproteins were fractionated; cholesterol in each of the 20 fractions was then quantified. The intake of BCE for 29 days significantly reduced the levels of small VLDL and large LDL cholesterols from 6.80 ± 2.7 mg to 3.72 ± 0.7 mg and from 28.1 ± 8.0 mg to 20.8 ± 4.5 mg, respectively (Table 2). In contrast, the TG level did not change with any lipoprotein (Supplementary Materials, Table S2).

## 3. Discussion

In the present study, we examined the effects of BCE on serum lipids in young healthy subjects. BCE intake did not change the serum TG level, but significantly decreased the total cholesterol level. Polyphenols such as anthocyanins have been reported to exhibit hypocholesterolemic effects [17,20,21,22,23]. Furthermore, Benn et al. reported that BCE powder containing 25% anthocyanins and 40% polyphenols reduced the serum levels of total cholesterol, but not of TGs, in mice fed a high-fat diet [15]. Our results are in agreement with those of the previous study. In this study, it is not clear why serum triglyceride levels did not change. Considering that the administration of BCE did not significantly reduce serum triglyceride levels, even in mice fed a high-fat diet, BCE may not be very effective in the absorption of triglycerides [15]. However, because it has been reported that the intake of BCE reduces the amount of triglyceride synthesized in the liver [15,19], it is possible that the synthesis of triglycerides in the liver is suppressed even in healthy subjects.

Regarding the mechanism of BCE-induced hypocholesterolemic effects, Kim et al. reported that anthocyanins in BCE increased the expression of LDL receptors in Caco-2 cells, resulting in a decrease in serum LDL cholesterol levels [24]. In the present study, the intake of BCE possibly increased the uptake of LDL by the LDL receptors; however, because the subjects in this study were young and healthy, the change in LDL cholesterol may have been difficult to detect. However, in the detailed profile of 20 fractions, the levels of small VLDL and large LDL cholesterols were significantly decreased, and it was expected that the serum level of total cholesterol would be lowered due to the enhanced cellular uptake of these lipoproteins. In vitro validation may be necessary in the future.

Polyphenols such as anthocyanins inhibit the absorption of cholesterol from the intestinal tract [25,26,27]. Furthermore, Nekohashi et al. reported that polyphenols inhibit the cholesterol absorption transporter Niemann-Pick C1-Like 1 expressed in human intestinal epithelial cells and prevent the increase in the concentration of food-derived cholesterol in the blood [28]. Although the inhibition of cholesterol absorption was not examined in this study, the inhibitory effect of anthocyanins on cholesterol has been reported [25,26,27], and it is highly possible that the BCE, being rich in anthocyanins, inhibited the absorption.

More cholesterol is synthesized in the body than is absorbed from food. Hydroxymethylglutaryl-CoA reductase (HMGR) is known to catalyze the rate-limiting step. Several polyphenols are known to inhibit HMG-CoA reductase, and the expression of HMGR is inhibited by BCE and blueberry anthocyanins [24,29]. Based on these reports, it is possible that the expression of HMGR was suppressed by the absorbed anthocyanins in this study.

Among LDL cholesterol, small, dense LDL cholesterol is more likely to be a direct cause of atherosclerosis because it is small enough to penetrate the walls of blood vessels and remains in the blood for a long time to be oxidized [30,31]. In fact, it has been reported that levels of small, dense LDL and very small, dense LDL are increased in patients with coronary artery disease, as determined using the GP-HPLC method in this study [32]. In this study, the VLDL cholesterol level was significantly decreased, and the LDL cholesterol levels tended to decrease. In addition, the levels of small, dense and very small, dense LDL did not decrease, but it was difficult to verify this because the subjects in this study were healthy and the small LDL cholesterol levels were not high at baseline. In this experiment, 12 young healthy subjects were administered BCE for approximately 1 month. In the future, if the study is conducted on a large scale over a longer period, a decrease in small, dense LDL may be observed. Moreover, Tahvonen et al. reported that the administration of blackcurrant seed oil to 15 healthy women for 4 weeks reduced serum LDL cholesterol levels [33]. In this study, the tendency for LDL cholesterol levels to decrease might have been due to the effects of fatty acids present in BCE.

We found that BCE has phytoestrogenic effects. In animal experiments, BCE has been shown to improve lipid abnormalities and vascular endothelial function [19,34,35]. In addition, BCE exhibited cosmetic effects in a menopausal rat model [36,37]. In this study, we targeted young, healthy women, but it is predicted that the daily intake of BCE from a young age before menopause will lead to the suppression of arteriosclerosis. These results suggest that the daily intake of BCE may have beneficial effects in women.

## 4. Materials and Methods

### 4.1. Materials and Reagents

The BCE powder, Active Cassis, a dietary supplement, was purchased from Just The Berries Limited, Palmerston North, New Zealand. One hundred grams of the powder consists of 35.0 g of total anthocyanins, 13.6 mg of carbohydrates, 0.5 g of fats, and 0.1 g of proteins, and yields 1650 kilojoules (394 Kcal) of energy [38]. A total of 230 mg BCE was filled in opaque hard capsules. No adverse events were observed with the use of the BCE powder. The powder is commercially available as a food material. The microbial and heavy metal content of the BCE powder is below the permissible values. Furthermore, the BCE powder was used within the expiration date, and no safety issues were noted.

### 4.2. Subjects

The subjects in this study were young, healthy females (*n* = 12, mean age 21 ± 2 years), students at Hirosaki University. They had typical heights, weights, and body mass index values (Table 3). The exclusion criteria were the presence of any disease, such as hypertension, hyperlipidemia, and diabetes; the use of a weight-reducing dietary regimen; or the consumption of any dietary supplements. All subjects were requested to complete a health and lifestyle questionnaire, and suitable subjects were requested to sign an informed consent form. The study protocol was approved by the Committee of Medical Ethics of Hirosaki University Graduate School of Health Sciences, Hirosaki, Japan (permission number: 2016-046 and date of approval: 20 March 2019). All participants provided informed consent.

### 4.3. Study Design

The subjects were administered two capsules daily for 28 days. Blood was collected one day before BCE intake (day 0) and on days 4 (day 4) and 29 (day 29) of BCE intake (Table 4). The blood samples were placed in blood collection tubes and the serum was separated by centrifugation at 1500× *g* for 10 min.

### 4.4. Biochemical Analysis of Serum Lipids

The lipoprotein and serum lipid profile data were obtained using GP-HPLC, LipoSEARCH^®^, which is a lipoprotein analysis service; the analysis was performed at Skylight Biotech, Akita, Japan. Briefly, cholesterol and triglycerides contained in the major fractions of lipoproteins (CM, VLDL, LDL, and HDL) and the 20 fractions (subclass defined by particle diameter, Table 5) were determined by GP-HPLC [39]. The major classes of lipoproteins are divided into 20 fractions by diameter. Fractions 1–2 are CM, fractions 3–7 are VLDL, fractions 8–13 are LDL, and fractions 14–20 are HDL [39]. In addition, each lipoprotein was classified into a subclass, the details of which are shown in Table 5.

### 4.5. Statistical Analysis

Results are expressed as the mean ± standard deviation. Graphs were generated using GraphPad Prism 7.0 ver. 7.03 (GraphPad Prism, San Diego, CA, USA). Statistically significant differences were determined using a paired one-way analysis of variance (ANOVA) with Bonferroni correction using the bell curve for Excel ver. 3.2 (Social Survey Research Information Co., Ltd., Tokyo, Japan). Results with *p*-values < 0.05 were considered statistically significant.

## 5. Conclusions

Serum dyslipidemia is a risk factor for atherosclerosis. In this study, we examined the effects of BCE on serum lipid levels in healthy young women as test subjects. There was no effect after 4 days of BCE intake, but the total serum cholesterol level was lowered after 29 days of intake. In addition, the VLDL cholesterol level was significantly lowered, and LDL cholesterol also showed a decreasing trend. It was difficult to detect the effect of LDL cholesterol in this study because the subjects were healthy. BCE inhibits the absorption of cholesterol, suppresses the expression of HMGR, and promotes the uptake of LDL by the LDL receptor; therefore, it is presumed that serum cholesterol decreased as a result of these synergistic effects. Furthermore, because this was a noncomparative and pilot study, it will be necessary to conduct randomized placebo-controlled studies for a longer period and with a larger number of individuals in the future to confirm the results.

## Figures and Tables

**Table 1:** Cholesterol concentration in the major classes of lipoproteins.

Major Class	0 Day (mg/dL)	4 Days (mg/dL)	29 Days (mg/dL)
CM	0.52 ± 0.3	0.57 ± 0.8	0.80 ± 1.0
VLDL	24.4 ± 7.2	21.4 ± 6.8	18.6 ± 4.7 *
LDL	87.9 ± 12.3	83.5 ± 21.2	82.8 ± 20.6
HDL	60.1 ± 9.5	59.5 ± 11.9	58.7 ± 11.6

Data are presented as the mean ± SD for 12 individuals. * *p* < 0.05 vs. 0 day.

**Table 2:** Cholesterol concentration in lipoprotein subclasses.

Subclass	0 Day (mg/dL)	4 Days (mg/dL)	29 Days (mg/dL)
Large VLDL	12.5 ± 4.4	11.3 ± 5.3	11.2 ± 4.3
Medium VLDL	5.04 ± 1.5	4.16 ± 1.3	4.66 ± 1.4
Small VLDL	6.80 ± 2.7	5.92 ± 1.0	3.72 ± 0.7 *
Large LDL	28.1 ± 8.0	26.8 ± 6.0	20.8 ± 4.5 *
Medium LDL	38.1 ± 5.3	36.9 ± 9.6	38.3 ± 9.6
Small LDL	15.0 ± 4.6	13.7 ± 3.8	17.1 ± 4.8
Very small LDL	6.79 ± 2.1	6.15 ± 1.5	6.61 ± 1.7
Very large HDL	4.86 ± 2.4	4.86 ± 2.6	4.07 ± 2.2
Large HDL	18.9 ± 6.9	18.7 ± 8.2	18.0 ± 7.9
Medium HDL	20.5 ± 2.2	20.0 ± 2.3	20.7 ± 2.4
Small HDL	10.9 ± 1.5	11.1 ± 1.5	11.3 ± 1.7
Very small HDL	4.91 ± 0.7	4.88 ± 0.7	4.59 ± 0.6

Data are presented as the mean ± SD for 12 individuals. * *p* < 0.05 vs. 0 day.

**Table 3:** Baseline characteristics of participants (*n* = 12).

Variable	Value
Age, years	21 ± 2
Height, cm	158.4 ± 14.4
Weight, kg	52.9 ± 17.1
Body mass index, kg/m^2^	21.1 ± 4.1

Values are expressed as means ± SD.

**Table 4:** Test schedule.

Days	0	1	2	3	4	⋯	28	29
Intake BCE (460 mg/day)		○	○	○	○	⋯	○	
Blood sampling	○				○	⋯		○

**Table 5:** Particle diameter in major classes and subclasses of lipoproteins.

Fraction No.	Particle Diameter (nm)	Subclass	Major Class
1	>90	−	CM (>80 nm)
2	75.0	−	
3	64.0	Large VLDL	VLDL (30–80 nm)
4	53.6		
5	44.5		
6	36.8	Medium VLDL	
7	31.3	Small VLDL	
8	28.6	Large LDL	LDL (16–30 nm)
9	25.5	Medium LDL	
10	23.0	Small LDL	
11	20.7		
12	18.6	Very small LDL	
13	16.7		
14	15.0	Very large HDL	HDL (8–16 nm)
15	13.5		
16	12.1	Large HDL	
17	10.9	Medium HDL	
18	9.8	Small HDL	
19	8.8	Very small HDL	
20	7.6		

CM, chylomicron; VLDL, very-low-density lipoprotein; LDL, low-density lipoprotein; HDL, high-density lipoprotein.

## Data Availability

The data that support the findings of this study are available from the corresponding author, upon reasonable request.

## Supplementary Materials

The following are available online, Table S1: Triglyceride concentration in major classes of lipoproteins; Table S2: Triglyceride concentration in lipoprotein subclasses.

## Sample Availability

Samples of the BCE powder are available from the authors.
