# Supplementary material for: Hypocholesterolemic Effect of Blackcurrant (Ribes nigrum) Extract in Healthy Female Subjects: A Pilot Study
Source: Molecules. 2021 Jul 4;26(13):4085. doi: 10.3390/molecules26134085 (PMC8272003; doi:10.3390/molecules26134085)
Supplement: Supplementary file 1 [file molecules-26-04085-s001.zip › molecules-1280284-supplementary.pdf]

Supplementary Materials

Hypocholesterolemic Effect of Blackcurrant (*Ribes nigrum*) Extract in Healthy Female Subjects: A Pilot Study

Naoki Nanashima [1]*, Kayo Horie [1], Maiko Kitajima [2], Shizuka Takamagi [2], Kasumi Mikami [2], Naoya In [2] and Toshiko Tomisawa [2]*

[1] Department of Bioscience and Laboratory Medicine, Hirosaki University Graduate School of Health Sciences, Hirosaki, Japan; nnaoki@hirosaki-u.ac.jp (N.N); k-horie@hirosaki-u.ac.jp (K.H.)

[2] Department of Nursing Sciences, Hirosaki University Graduate School of Health Sciences, 66-1 Hon-cho, Hirosaki, Aomori 036-8564, Japan; kitajima@hirosaki-u.ac.jp (M.K.); takamagi@hirosaki-u.ac.jp (S.T.); k-mikami@hirosaki-u.ac.jp (K.M.); in1105@hirosaki-u.ac.jp (N.I.); tmtott@hirosaki-u.ac.jp (T.T.)

* Correspondence: nnaoki@hirosaki-u.ac.jp (N.N.); tmtott@hirosaki-u.ac.jp (T.T.)

Table S1. Triglyceride concentration in the major classes of lipoproteins.

Classes	0 day (mg/dL)	4 days (mg/dL)	29 days (mg/dL)
CM	3.20 ± 2.7	4.64 ± 7.0	6.32 ± 9.2
VLDL	45.1 ± 32.7	45.9 ± 46.0	44.0 ± 41.9
LDL	17.9 ± 3.79	16.3 ± 3.7	16.6 ± 3.4
HDL	13.0 ± 3.8	11.4 ± 4.9	11.6 ± 5.1

Data represent the means ± SD for 12 individuals.

Table S2. Triglyceride concentration in lipoprotein subclasses.

Subclasses	0 day (mg/dL)	4 days (mg/dL)	29 days (mg/dL)
Large VLDL	30.1 ± 25.5	32.7 ± 39.4	31.1 ± 35.6
Medium VLDL	10.7 ± 5.2	9.23 ± 4.9	9.04 ± 4.7
Small VLDL	4.32 ± 1.2	3.99 ± 1.2	3.86 ± 1.1
Large LDL	6.60 ± 1.3	6.10 ± 1.4	6.01 ± 1.3
Medium LDL	6.58 ± 1.3	6.03 ± 1.2	6.35 ± 1.2
Small LDL	3.08 ± 1.3	2.72 ± 0.7	2.86 ± 0.6
Very small LDL	1.68 ± 0.7	1.49 ± 0.5	1.39 ± 0.5
Very large HDL	1.12 ± 0.6	0.92 ± 0.4	1.04 ± 0.4
Large HDL	4.25 ± 1.7	3.44 ± 1.1	3.75 ± 1.2
Medium HDL	4.21 ± 1.3	3.74 ± 1.9	3.90 ± 2.0
Small HDL	2.17 ± 0.7	2.09 ± 1.2	1.86 ± 1.1
Very small HDL	1.28 ± 0.4	1.24 ± 0.6	1.05 ± 0.5

Data represent the means ± SD for 12 individuals.
